# Recurrent Pure Calcite Urolithiasis Confirmed by Endoscopic Removal and Infrared Spectroscopy in a Malnourished Anorectic Female

**DOI:** 10.1089/cren.2016.0036

**Published:** 2016-04-01

**Authors:** Frederikke Eichner Christiansen, Kim Hovgaard Andreassen, Palle Jörn Sloth Osther

**Affiliations:** Department of Urology, Urological Research Center, Lillebaelt Hospital, University of Southern Denmark, Fredericia, Denmark.

## Abstract

Often when calcite is found as a component of urinary calculi, they are considered false calculi or artifacts. We present a case of true calcite urolithiasis. The stone material was removed percutaneously from a severely malnourished anorectic woman and analyzed by infrared spectroscopy (IRS). In addition, calcite urolithiasis was confirmed in several recurrent stone events by IRS. Laxative abuse with magnesium oxide was believed to be the underlying cause of stone formation, and ammonium chloride given as one weekly dose turned out to be effective for stone prevention.

## Introduction

As part of the metabolic work-up in patients with kidney calculi, stone analysis should be performed at least once in all stone formers. The preferred analytical methods include infrared spectroscopy (IRS) and X-ray diffraction. The majority of stones are of mixed composition. In humans the most frequent stone components include calcium oxalate (whewellite [COM] and weddellite [COD]) and calcium phosphate (apatite and brushite). Calcite is rarely considered a true component of a kidney calculus, being present in only 0.01% to 0.25% of all stones, and usually mixed with other components.^1–3^ Calcite is a crystal polymorph of calcium carbonate (CaCO_3_). Other crystal polymorphs with the same chemical composition include aragonite and vaterite. Calcite is a common earth mineral and the principal constituent in lime stone and marble, common in human pancreatic and salivary lithiasis and in equine urolithiasis, but rare in human urolithiasis.^1^ Calcite stones presented by patients as spontaneously passed are often artifactual or factitious. We report a 42-year-old malnourished woman with anorexia nervosa forming recurrent pure calcite stones that were removed endoscopically and analyzed by IRS at several occasions.

## Case Report

**FIG. 1.**
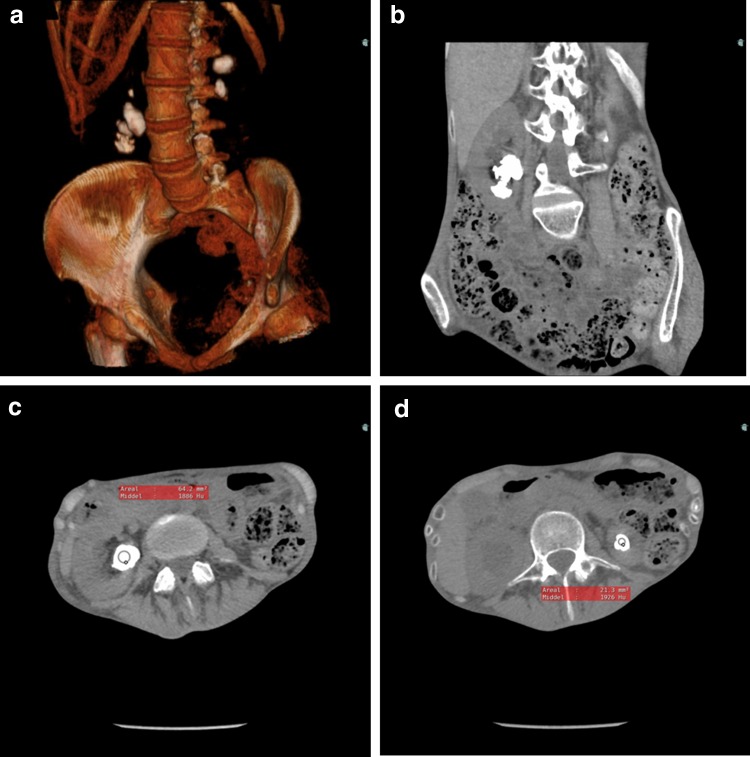
Three-dimensional reconstruction **(a)**, coronal **(b)**, and axial **(c, d)** CT images of bilateral calcite-nephrolithiasis in a malnourished anorectic woman. The stones were homogeneous with Hounsfield units around 1900.

A 42-year-old woman with anorexia since adolescence and a 5-year history of recurrent nephrolithiasis was presented because of accumulated renal colics, migraine, and nausea. Noncontrast CT imaging showed bilateral nephrolithiasis and hydronephrosis. The stones were homogeneous and had a Hounsfield unit around 1900 (Fig. 1). She was severely malnourished with a BMI of 12.0 $\frac { kg }  { m^2 }$ (height: 162 cm, weight: 31.6 kg) when she was first presented. The patient initially refused abuse of laxantia, but later in the disease course she admitted to have had a long-standing intake of magnesium oxide. Blood samples at admission showed low albumin, low total plasma calcium, low magnesium, and normal potassium, sodium, phosphate, creatinine, and urea. Urine pH was 7.0. Urine dipstick was positive for blood, leucocytes, and protein. Urine culture was negative. A 24-hour urine analysis showed a volume of 1865 mL, low citrate (0.4 mmol/24 hour), normal oxalate (211 μmol/24 hour), moderately high calcium (5 mmol/24 hour), and low creatinine (5 mmol/24 hour). Renography showed a left/right ratio of 22%/78%.

**FIG. 2.**
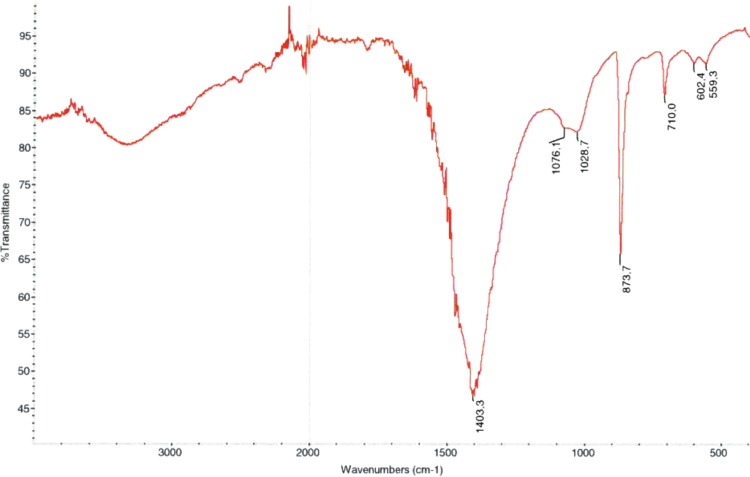
Infrared spectroscopic analysis of the right-sided kidney stone revealing pure calcite.

The patient underwent bilateral percutaneous stone removal (PCNL). Stone analysis from both sides using IRS revealed pure calcite (CaCO_3_) (Fig. 2). The patient had several recurrences within the next 3 years, and underwent repeated flexible retrograde intrarenal surgery (RIRS) and extracorporeal shockwave lithotripsy (SWL) procedures with subsequent IRS stone analyses of the extracted and post-SWL-cleared stones, confirming pure calcite. Due to low urinary citrate excretion, moderately high calcium excretion, high urine pH, and normal systemic acid–base status, incomplete renal tubular acidosis was initially suspected. Oral potassium citrate therapy failed to prevent frequent recurrences, however. Because the patient had high urine pH (7.0), a new treatment strategy with oral ammonium chloride (800 mg three times a day, 1 day per week) was initiated, based on the assumption that calcite crystallization may be promoted at high urine pH, as indicated by a study of Gault and colleagues.^4^ On this treatment, the patient has not formed new stones during an observation period of 23 months.

## Discussion

Often when calcite is found as a component of urinary calculi, they are considered false calculi or artifacts.^3^ In this case, renal calculi were obtained on several occasions directly from the kidney by PCNL and RIRS for analysis, similar to a case previously reported in a male with acromegaly.^5^ In that case, the patient had a mixture of stones containing COM, COD, and apatite in addition to fragments composed of calcite. In an Egyptian study of pediatric nephrolithiasis, calcite was found in 10% of cases; however, the underlying causes of calcite stone formation were not discussed.^6^ Studies have shown that anorexia nervosa may increase the risk of nephrolithiasis, possibly because of a low urinary output.^7^ In previous case reports on urolithiasis in anorexia nervosa, calculi most frequently were composed of calcium oxalate or ammonium urate.^7^ The underlying cause of calcite urolithiasis is unclear. It has been shown that a neutral pH (7) promotes a transformation of amorphous calcium carbonate (ACC) into calcite through a dissolution/reprecipitation mechanism.^8^ Furthermore, magnesium increased the stability of ACC and favored the formation of calcite.^8^ The observations on the effect of pH may be why urinary acidification with ammonium chloride was effective in our case. The cause of high urine pH in the present case is unknown. It is unlikely to believe that it was because of impaired urinary acidification, since this usually results in calcium phosphate stone formation. Magnesium oxide abuse, however, may have resulted in high urine pH and magnesium levels, which could have created a urinary milieu in favor of calcite crystallization. If known early in the course of the present stone disease, magnesium oxide abuse of course should have been discontinued, although this may be difficult to control in anorectic individuals.^7^ In this case of mixed COM, COD, apatite, and calcite urolithiasis in a patient with acromegaly, the urine pH was quite acidic (pH = 5.2), which is not consistent with calcium carbonate crystal formation.^4^ It was conceivable, however, that the particular calcite stone was formed in an obstructed part of the kidney, in which the urinary milieu may have been alkaline.^4^

## Conclusion

Calcite stones are not always artifactual or factitious. Severe malnutrition and abuse of magnesium-containing laxantia may be involved in the pathogenesis of urinary calcite stone formation. Urinary acidification with ammonium chloride given as one weekly dose may be effective for stone prevention.

## Abbreviations Used

ACC: amorphous calcium carbonate
BMI: body mass index
CaCO_3_: calcium carbonate
CT: computed tomography
IRS: infrared spectroscopy
PCNL: percutaneus stone removal
RIRS: retrograde intrarenal surgery
SWL: extracorporeal shockwave lithotripsy
